# mOWL: Python library for machine learning with biomedical ontologies

**DOI:** 10.1093/bioinformatics/btac811

**Published:** 2022-12-19

**Authors:** Fernando Zhapa-Camacho, Maxat Kulmanov, Robert Hoehndorf

**Affiliations:** Computer, Electrical and Mathematical Sciences & Engineering Division (CEMSE), Computational Bioscience Research Center (CBRC), King Abdullah University of Science and Technology, Thuwal 23955, Saudi Arabia; Computer, Electrical and Mathematical Sciences & Engineering Division (CEMSE), Computational Bioscience Research Center (CBRC), King Abdullah University of Science and Technology, Thuwal 23955, Saudi Arabia; Computer, Electrical and Mathematical Sciences & Engineering Division (CEMSE), Computational Bioscience Research Center (CBRC), King Abdullah University of Science and Technology, Thuwal 23955, Saudi Arabia

## Abstract

**Motivation:**

Ontologies contain formal and structured information about a domain and are widely used in bioinformatics for annotation and integration of data. Several methods use ontologies to provide background knowledge in machine learning tasks, which is of particular importance in bioinformatics. These methods rely on a set of common primitives that are not readily available in a software library; a library providing these primitives would facilitate the use of current machine learning methods with ontologies and the development of novel methods for other ontology-based biomedical applications.

**Results:**

We developed mOWL, a Python library for machine learning with ontologies formalized in the Web Ontology Language (OWL). mOWL implements ontology embedding methods that map information contained in formal knowledge bases and ontologies into vector spaces while preserving some of the properties and relations in ontologies, as well as methods to use these embeddings for similarity computation, deductive inference and zero-shot learning. We demonstrate mOWL on the knowledge-based prediction of protein–protein interactions using the gene ontology and gene–disease associations using phenotype ontologies.

**Availability and implementation:**

mOWL is freely available on https://github.com/bio-ontology-research-group/mowl and as a Python package in PyPi.

**Supplementary information:**

Supplementary data are available at *Bioinformatics* online.

## 1 Introduction

The use of machine learning in bioinformatics has been rapidly increasing, and computational power and data availability enabled substantial advances in many areas of bioinformatics through machine learning (Li *et al.*, 2019). A crucial aspect of the success of machine learning methods was the development of software tools and machine learning libraries such as TensorFlow (Abadi *et al.*, 2016) and PyTorch (Paszke *et al.*, 2019).

Ontologies include rich and structured knowledge about a domain of discourse. They are widely used in biology and biomedicine with more than 1000 ontologies available in BioPortal (Whetzel *et al.*, 2011). Ontologies are used to facilitate tasks such as data integration across databases, annotation of biological entities, data access and analysis, and providing background knowledge of a domain (Hoehndorf *et al.*, 2015). Background knowledge is provided by ontologies through machine-readable axioms. For example, the gene ontology (GO) (Ashburner *et al.*, 2000) contains around 50 000 classes and over 100 000 logical axioms.

Some recent machine learning methods can utilize logical axioms to improve domain-specific tasks by utilizing background knowledge, in particular through ontology embeddings. An ontology embedding is a function that maps ontology entities (classes, instances and relations) to the $R^{n}$ while preserving some of the knowledge in the logical axioms of the ontology. Ontology embedding methods can be divided into graph-based methods (i.e. projecting logical axioms onto a graph), syntactic methods (utilizing the axioms directly) and semantic methods (generating semantic interpretations from the axioms) (Kulmanov *et al.*, 2021).

Ontology embeddings have shown to be useful across different problems, and in particular in biological and biomedical problems that rely on data represented through ontologies. Ontology embeddings can be used directly to predict associations between entities annotated with ontologies, such as gene–disease associations (GDAs) based on the relations between their phenotype annotations (Smaili *et al.*, 2019), they can be used to provide features for larger machine learning models (Hinnerichs and Hoehndorf, 2021), or they can enable zero-shot predictions (Kulmanov and Hoehndorf, 2022).

We developed mOWL, a Python library for machine learning with Web Ontology Language (OWL) ontologies. The purpose of mOWL is to serve as a reference implementation for ontology embeddings and to enable the implementation of new ontology embedding methods. For this purpose, mOWL provides functionality to access information in ontologies and to reason over ontologies, and it provides methods to access biomedical databases that rely on ontologies for annotation.

## 2 Implementation

mOWL has been designed to handle input in OWL format and generate embeddings that can be used to classify entities or predict new axioms. mOWL consists of components for (i) ontology management, normalization and reasoning, interfacing with the OWL API (Horridge and Bechhofer, 2011) and automated reasoners; (ii) ontology transformation, including methods for projecting ontologies into graphs, text corpora or other formats used as precursor to machine learning; (iii) embedding generation by interfacing with the knowledge graph embedding library PyKEEN (Ali *et al.*, 2021) and other approaches implemented in Python; and (iv) embedding post-processing, including axiom inference, node classification, evaluation and visualization. Supplementary Figure S1 provides an overview of the components (i)–(iv).

mOWL has been developed to be used as a Python package. However, mOWL interfaces with the OWL API to enable ontology processing and automated reasoning. The OWL API is implemented in Java, and we use JPype (Nelson and Scherer, 2020) to bind Python and the Java Virtual Machine, enabling access to Java classes and methods from Python. mOWL therefore provides access to the entire OWL API through a Python interface.

## 3 Use cases

mOWL can be used to implement at least two types of models. The first relies on generating ontology embeddings to induce background knowledge in machine learning methods. Examples of these methods can be found in Supplementary Tables S1–S3 where we tested the methods implemented in mOWL on the tasks of predicting protein–protein interactions and GDAs. For both tasks, we used three types of methods: graph-based, syntactic and semantic. In most evaluation metrics and across both tasks, we find that graph-based ontology embedding methods perform better than other methods.

The second type of model for which mOWL can be used consists of using the background knowledge in ontologies to constrain the learning objective and imposing a structure on representations generated by a machine learning method. In these applications, it becomes possible to perform some kind of logical operations on representations of machine learning methods, and use these, for example, for zero-shot predictions. An example of such a method can be found in mOWL where we provide an implementation of DeepGOZero (Kulmanov and Hoehndorf, 2022), a model that performs zero-shot predictions of protein functions.

In addition to the use cases enabled by mOWL, through the direct interfacing with the OWL API and JPype, mOWL is also very fast in comparison to some other implementations of ontology embedding methods. We provide a comparison in Supplementary Table S4.

## 4 Conclusion

mOWL is a library intended to generate ontology embeddings. Ontology embeddings are broadly applicable to incorporating background knowledge in biological machine learning methods due to the large number of biomedical ontologies used in bioinformatics. mOWL provides implementation of state-of-the-art methods, datasets, OWL API integration and functionalities to manage ontologies. mOWL also provides functionalities for the implementation of novel methods.

## Supplementary Material

btac811_Supplementary_Data

## Data Availability

Our outcome is a software package. We provide some benchmark datasets that we used to generate our results and are accessible from the package itself. However, the data can also be accessed from this link: https://bio2vec.cbrc.kaust.edu.sa/data/mowl/.
