# Supplementary material for: mOWL: Python library for machine learning with biomedical ontologies
Source: Bioinformatics. 2022 Dec 19;39(1):btac811. doi: 10.1093/bioinformatics/btac811 (PMC9848046; doi:10.1093/bioinformatics/btac811)
Supplement: btac811_Supplementary_Data [file btac811_supplementary_data.zip › supplementary.pdf]

mOWL: Python library for machine learning with biomedical ontologies
Supplementary Material

Fernando Zhapa-Camacho Maxat Kulmanov
Robert Hoehndorf

November 23, 2022

In this supplementary material we show the results of methods implemented in mOWL across different datasets. In each section we detail how datasets were constructed. In terms of methods, we have classified them into three categories: graph-based methods, syntactic and semantic.

Graph based methods are split into two groups: random-walk based and knowledge graph embedding methods. In the case of knowledge graph embedding methods we have performed hyperparameter optimization (HPO) as follows:

- TransE:
  - vector size: $[50, 100, 150, 200, 300, 400]$
  - epochs: $[20, 40, 60]$
  - learning rate: $[10^{-4}, 10^{-3}, 10^{-2}, 10^{-1}]$
  - batch size: $[2048, 4096, 8192]$
- DistMult:
  - vector size: $[50, 100, 150, 200, 300, 400]$
  - epochs: $[20, 40, 60]$
  - learning rate: $[10^{-4}, 10^{-3}, 10^{-2}, 10^{-1}]$
  - batch size: $[2048, 4096, 8192]$
- ConvKB:
  - vector size: $[100, 200, 300]$
  - epochs: $[40, 50, 60, 70]$
  - learning rate: $[10^{-4}, 10^{-3}, 10^{-2}, 10^{-1}]$
  - batch size: $[1024, 2048, 4096, 8192]$

– hidden dropout rate: $[0.0, 0.1, 0.2, 0.3]$
– number of filters: $[50, 100, 200, 400]$

The algorithm followed for HPO is called Tree-structured Parzen Estimator provided by Optuna library.

1 Protein-protein interaction results across several models

In this section we find experiments on the protein–protein interaction prediction task using methods found in mOWL either by direct implementation or interfacing with other libraries (such as PyKEEN). The data used for this experiments consists of the Gene Ontology released on 20-10-2021 (downloaded from http://purl.obolibrary.org/obo/go.owl) and protein interaction data found in String Database version 11.5 (downloaded from https://stringdb-static.org/download/protein.links.v11.5.txt.gz). Particularly, the data used was that related to yeast species. In mOWL, it is available as a built-in dataset called `PPIYeastDataset` that consists of three ontologies: training, validation and testing. Protein interaction data was randomly split 90:5:5 across training, validation and testing ontologies and Gene Ontology functional annotations of proteins is part of the training ontology only. Protein interactions are represented as an axiom of the form $protein_1 \sqsubseteq interacts\_with.protein_2$.

Method		Hits@1		Hits@10		Hits@100		Mean rank		Rank AUC	
		R	F	R	F	R	F	R	F	R	F
**Graph based:**											
DL2Vec +	DeepWalk	0.000	0.000	0.040	0.056	0.399	0.427	269.310	258.390	0.950	0.960
	Node2Vec	0.000	0.000	0.000	0.000	0.014	0.014	664.930	664.810	0.890	0.890
	TransE	0.000	0.000	**0.101**	**0.353**	0.567	**0.827**	**203.993**	**139.965**	**0.966**	**0.977**
	DistMult	0.005	0.011	0.090	0.243	0.492	0.685	254.412	198.026	0.957	0.967
	ConvKB	0.006	0.035	0.064	0.333	0.521	0.810	246.425	177.414	0.959	0.970
OWL2Vec* +	DeepWalk	0.000	0.000	0.005	0.007	0.269	0.280	356.010	349.450	0.940	0.940
	Node2Vec	0.000	0.000	0.000	0.000	0.016	0.016	693.330	693.160	0.880	0.880
	TransE	0.000	0.000	0.082	0.352	0.549	0.805	221.963	153.327	0.963	0.974
	DistMult	0.006	0.017	0.087	0.242	0.487	0.671	272.876	216.999	0.954	0.964
	ConvKB	0.002	**0.063**	0.042	0.346	0.506	0.819	234.427	158.849	0.961	0.973
**Syntactic:**											
Onto2Vec		**0.007**	0.014	0.069	0.120	0.528	0.609	282.900	251.240	0.950	0.960
OPA2Vec		**0.007**	0.013	0.066	0.106	0.550	0.605	288.730	264.480	0.950	0.960
**Semantic:**											
ELEmbeddings		0.006	0.013	0.059	0.120	0.417	0.622	296.194	234.919	0.951	0.961
ELBoxEmbeddings		0.004	0.007	0.010	0.318	**0.588**	0.820	216.282	160.535	0.964	0.974

Table 1: Protein-protein interaction prediction across different methods.

2 Gene-disease association results across several models

In this section we show results on prediction of gene–disease association on two species: mouse and human. For this experiment, we used phenotypic annota-

tions of genes and diseases and computed associations based on phenotypic similarity. For genes annotations we used the *Mouse/Human Orthology with Phenotype Annotations* document downloaded from http://www.informatics.jax.org/downloads/reports/HMD_HumanPhenotype.rpt. Disease annotations were obtained from the *HPO annotations for rare diseases* document dowloaded from http://purl.obolibrary.org/obo/hp/hpoa/phenotype.hpoa. These annotations were added to the *Unified Phenotype Ontology*(uPheno) to build the training ontology.

Futhermore, gene-disease associations were obtained from the *Associations of Mouse Genes with DO Diseases* file downloaded from http://www.informatics.jax.org/downloads/reports/MGI_DO.rpt. From this file, associations for human and mouse were extracted and each of them were randomly split 80:10:10, added to the training ontology and created the validation and testing ontologies, respectively. In this way, we created the `GDAHumanDataset` and `GDAMouseDataset` built-in datasets. For the semantic models over the description language $\mathcal{EL}$, it was necessary to remove complex axioms from the training ontology. This resulted in the `GDAHumanELDataset` and `GDAMouseELDataset` datasets.

2.1 Mouse

Method		Hits@1		Hits@10		Hits@100		Mean rank		Rank AUC	
		R	F	R	F	R	F	R	F	R	F
**Graph based:**											
DL2Vec +	DeepWalk	0.000	0.000	0.000	0.000	0.032	0.032	842.330	842.120	0.901	0.901
	Node2Vec	0.000	0.000	0.000	0.000	0.120	0.120	763.600	762.580	0.911	0.911
	TransE	0.008	0.329	0.558	**0.692**	**0.800**	**0.804**	**322.958**	**318.337**	**0.963**	**0.964**
	DistMult	**0.087**	0.217	0.467	0.529	0.700	0.700	695.381	691.615	0.919	0.920
	ConvKB	0.050	0.263	0.450	0.567	0.708	0.708	540.323	535.894	0.937	0.938
OWL2Vec* +	DeepWalk	0.000	0.000	0.000	0.000	0.012	0.012	1197.940	1197.580	0.859	0.859
	Node2Vec	0.000	0.000	0.000	0.000	0.044	0.044	949.740	948.800	0.889	0.889
	TransE	0.021	**0.342**	**0.604**	0.667	0.758	0.762	344.171	340.042	0.961	0.961
	DistMult	0.046	**0.342**	0.592	0.658	0.725	0.725	996.840	992.744	0.883	0.884
	ConvKB	0.000	0.308	0.429	0.608	0.721	0.725	616.617	611.154	0.928	0.929
**Syntactic:**											
Onto2Vec		0.004	0.004	0.046	0.050	0.475	0.492	1365.071	1362.390	0.839	0.839
OPA2Vec		0.000	0.000	0.029	0.029	0.450	0.450	1557.319	1554.821	0.816	0.816
**Semantic:**											
ELEmbeddings		0.019	0.024	0.105	0.119	0.315	0.323	2292.836	2290.353	0.728	0.729
ELBoxEmbeddings		0.016	0.194	0.332	0.380	0.426	0.429	2257.307	2254.620	0.732	0.733

Table 2: Mouse gene-disease association prediction across different methods.

2.1.1 Human

Method		Hits@1		Hits@10		Hits@100		Mean rank		Rank AUC	
		R	F	R	F	R	F	R	F	R	F
**Graph based:**											
DL2Vec +	DeepWalk	0.000	0.000	0.009	0.009	0.130	0.131	2413.580	2412.610	0.714	0.714
	Node2Vec	0.000	0.000	0.007	0.007	0.174	0.174	2530.000	2528.800	0.700	0.700
	TransE	0.007	0.125	0.235	0.385	**0.454**	**0.460**	**1699.151**	**1695.330**	**0.799**	**0.799**
	DistMult	0.027	0.098	0.246	0.360	0.432	0.434	1931.352	1928.061	0.771	0.771
	ConvKB	0.014	0.088	0.184	0.311	0.393	0.393	1975.406	1971.498	0.766	0.766
OWL2Vec* +	DeepWalk	0.000	0.000	0.000	0.000	0.033	0.033	2803.980	2803.170	0.667	0.667
	Node2Vec	0.000	0.000	0.001	0.001	0.068	0.068	2789.140	2788.210	0.669	0.669
	TransE	0.014	0.085	0.174	0.287	0.388	0.390	1975.922	1972.496	0.766	0.766
	DistMult	0.009	**0.170**	**0.278**	**0.425**	0.435	0.437	2221.648	2217.627	0.736	0.737
	ConvKB	0.004	0.081	0.111	0.264	0.363	0.365	2193.811	2189.135	0.740	0.740
**Syntactic:**											
Onto2Vec		0.001	0.011	0.023	0.025	0.216	0.219	2349.420	2347.290	0.721	0.721
OPA2Vec		0.001	0.001	0.014	0.016	0.187	0.193	2550.080	2548.150	0.697	0.698
**Semantic:**											
ELEmbeddings		**0.028**	0.113	0.250	0.373	0.408	0.409	2619.938	2616.655	0.690	0.690
ELBoxEmbeddings		0.004	0.120	0.194	0.323	0.363	0.365	2455.524	2451.806	0.709	0.710

Table 3: Human gene-disease association prediction across different methods.

3 Time comparison for OWL2Vec* projection method between original and mOWL implementation.

Parameters			Time for GO Slim Yeast (s)		Time for GO (s)	
**OT**	**BT**	**IL**	**OWL2Vec***	**mOWL**	**OWL2Vec***	**mOWL**
Yes	Yes	Yes	1.686	**0.042**	210.690	**8.490**
Yes	Yes	No	1.255	**0.125**	125.580	**2.450**
Yes	No	Yes	1.697	**0.069**	211.880	**6.890**
Yes	No	No	1.201	**0.005**	107.980	**1.670**
No	Yes	Yes	2.605	**0.028**	667.120	**11.140**
No	Yes	No	1.913	**0.017**	569.510	**4.530**
No	No	Yes	2.604	**0.025**	645.770	**10.480**
No	No	No	1.942	**0.007**	549.690	**3.530**

Table 4: Time comparison between mOWL and original implementations of the OWL2Vec* projection rules. Some of the parameters from the original implementation are: only taxonomy (OT), bidirectional taxonomy (BT), and include literals (IL). Tests were done with the Gene Ontology (GO) and a subset (GO Slim Yeast) of GO.

4 General representation of workflows in mOWL

In the Figure 1 we show how mOWL can be used to implement or access to different tools existing in the literature. As mentioned in the main document, the embeddings generated from an ontology can be of three types: graph-based,

syntactic or semantic. We show some reduced algorithms to show how the mOWL can be used to implement embeddings methods. Detailed examples can be found in the documentation website: https://mowl.readthedocs.io/en/latest/examples/index.html and can be downloaded as Jupyter notebooks.

4.1 Graph-based:

For this group of methods, mOWL provides several *projectors*, that will transform the ontology into a graph. Each projector has its own projection rules. Two particular examples of projectors are: `DL2VecProjector` and `OWL2VecStarProjector`.

After obtaining a graph, there are at least two ways of generating embeddings: using random walks or knowledge graph embedding methods. In the case of DL2Vec, we could implement it by following the algorithm:

Algorithm 1 Implementation of DL2Vec in mOWL

Require: $ontology\_file.owl$
Ensure: $ontology = ontology\_file.owl$
  $graph \leftarrow DL2VecProjector(ontology)$
  $walks \leftarrow DeepWalk(graph)$
  $embeddings = Word2Vec(walks)$

mOWL provides the classes `DL2VecProjector` and `DeepWalk`. `Word2Vec` is used from external source.

4.2 Syntactic:

In this category, we have grouped the methods that encode ontology entities using the textual information existing on it. To implement tools such as Onto2Vec, we could follow the following algorithm:

Algorithm 2 Implementation of Onto2Vec in mOWL

Require: $ontology\_file.owl$
Ensure: $ontology = ontology\_file.owl$
  $reasoner = MOWLReasoner(ontology)$
  $new\_axioms = reasoner.infer\_subclass\_axioms()$
  $ontology.add(new\_axioms)$
  $new\_axioms = reasoner.infer\_equivalent\_class\_axioms()$
  $ontology.add(new\_axioms)$
  $new\_axioms = reasoner.infer\_disjoint\_class\_axioms()$
  $ontology.add(new\_axioms)$
  $corpus = extract\_axiom\_corpus(ontology)$
  $embeddings = Word2Vec(corpus)$

mOWL provides the class `MOWLReasoner` and the methods `infer_subclass_axioms`, `infer_equivalent_class_axioms`, `infer_disjoint_axioms` and `extract_axiom_corpus`.

4.3 Semantic:

In this category, the model is designed in such a way that encodes the semantics of the axioms. An example of this methods is ELEmbeddings, which proposes a model to encode the semantics of the $\mathcal{EL}$ description logic. In mOWL, we provide the necessary methods to work with these kind of models. We can implement ELEmbeddings as follows:

Algorithm 3 Implementation of Onto2Vec in mOWL

Require: $ontology\_file.owl$

Ensure: $ontology = ontology\_file.owl$

  $el\_normal\_forms = ELDataset(ontology)$

  $el\_model = ELEmbeddings(el\_normal\_forms)$

  $el\_model.train()$

---

mOWL provides the class `ELDataset` and `ELEmbeddings`.

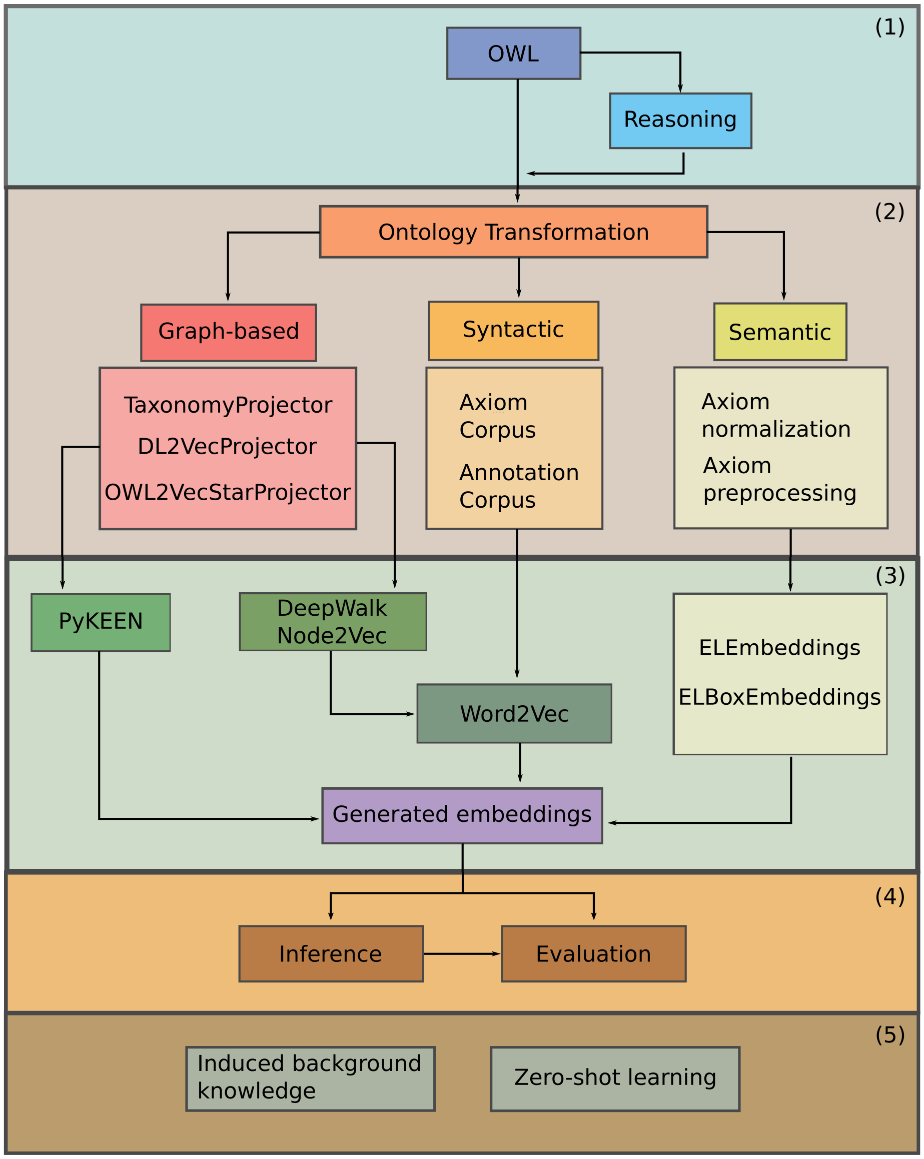

Figure 1: Different components existing in mOWL. (1) includes the ontology creation, modification and reasoning. (2) represent the graph-based and syntactic extraction information functionalities as well as the preprocessing steps needed for semantic models. (3) incorporates the embedding process that can be done with either graph-based, syntactic or semantic methods. (4) consists of the evaluation and inference modules for the generated embeddings. (5) is not a component of mOWL but shows the types of models that can be implemented based on how the background knowledge is used.
